# Weekly Versus Three-Weekly Administration of Paclitaxel as Neoadjuvant Chemotherapy in HER2 Negative, Stage III Breast Cancer: A Comparison of Treatment Responses

**DOI:** 10.7759/cureus.79636

**Published:** 2025-02-25

**Authors:** Shourov Biswas, Md Masudur Rahman, Sarwar Alam, Mostafa S Haque, Ekram B Faruq, Mohammad J Shams, Soma Banerjee

**Affiliations:** 1 Department of Clinical Oncology, Bangabandhu Sheikh Mujib Medical University, Dhaka, BGD

**Keywords:** breast cancer, her2 negative, neoadjuvant chemotherapy, stage iii breast cancer, weekly paclitaxel

## Abstract

Background and aim: Neoadjuvant chemotherapy with doxorubicin (A) and cyclophosphamide (C) followed by taxane (T) (AC followed by T) is one of the standard regimens in locoregionally advanced breast cancer. Paclitaxel, a taxane, can be given either in a three-weekly or a weekly schedule. The aim of this study was to compare the efficacy in terms of the clinical and pathological response of three-weekly paclitaxel with weekly paclitaxel in the treatment of human epidermal growth factor receptor 2 (HER2)-negative, stage III breast cancer.

Materials and methods: A quasi-experimental study was conducted from April 2022 to October 2023 in two centers in Dhaka, Bangladesh. Sixty-six patients were enrolled and divided equally into two arms. Arm-A patients received four cycles of AC followed by paclitaxel (175 mg/m^2^)three-weekly for four cycles. Patients in Arm-B received four cycles of AC followed by paclitaxel (80 mg/m^2^) weekly for 12 weeks. Patients were evaluated before, during, and after the completion of the chemotherapy to assess clinical outcomes and were assessed for the pathological response after surgical management.

Observations and results: Pathological complete response (pCR) was achieved in 15 (25%) patients. Four patients (13.33%) in Arm A and 11 patients (36.66%) in Arm B had pCR. The difference was statistically significant (p 0.037). The p-value was 0.219 for response at the primary site and 0.13 for response in axillary disease. In both arms, patients with triple-negative receptor status had increased pCR (22.22% or two patients out of nine in Arm A, and 85.71% or six patients out of seven in Arm B) in comparison to hormone receptor (estrogen receptor (ER) and/or progesterone receptor (PR)) positive tumors (9.52% or two patients out of 21 in Arm A, and 21.74% or five patients out of 23 in Arm B). However, these differences were not significant.

Conclusion: After three-weekly administrations of four cycles of AC, the weekly administration of paclitaxel was found to be more effective in comparison to three-weekly administration of paclitaxel.

## Introduction

According to the Global Cancer Incidence, Mortality and Prevalence (GLOBOCAN) database, breast cancer was the leading cancer in females worldwide in 2022 [1]. It accounted for almost 7% of total cancer mortality, with more than two million new cases in that same year. Its incidence is on the rise in Asia. More than a million new cases (45.4% of new cases worldwide) occurred in Asia in 2022 alone [1,2]. In Bangladesh, it is the most common in terms of incidence and second most common in terms of cancer-related mortality in females [3].

Although most patients in Western countries are diagnosed early, in less developed countries, about 60% of patients have locally advanced or metastatic disease at the time of diagnosis [4]. The scenario is somewhat similar in Bangladesh. As a vast majority of the patients of Bangladesh present with locally advanced disease, it is vital to address this group of patients [5]. Neoadjuvant chemotherapy is the standard approach in most of these patients. It is administered to aid surgical resectability. It also plays a vital role in preventing micrometastasis. In addition to these, neoadjuvant chemotherapy also provides information about the intrinsic chemosensitivity of the tumor, which is vital in determining further management [6]. Multiple prospective clinical trials demonstrated the prognostic value of response to neoadjuvant therapy [7,8].

The absence of invasive carcinoma from both breast and lymph nodes after neoadjuvant chemotherapy is termed as the pathological complete response (pCR). It is stated that ypT0N0 is the more stringent definition for it, but ypTisN0 is also acceptable as it does not affect the long-term outcome significantly [9]. A pathological complete response to neoadjuvant chemotherapy is associated with significant improvement in disease-free survival and overall survival for individual patients [9]. This association varies depending upon the luminal subtypes. A meta-analysis by Cortazar et al. confirmed the reproducible prognostic value of pCR [10]. Studies have shown that pCR is the most significant surrogate marker of overall survival and disease-free survival [6,10].

Paclitaxel is a common drug used in neoadjuvant settings. It can be administered in a three-weekly or weekly schedule. In Bangladesh, weekly paclitaxel is being practiced less frequently in comparison to three-weekly schedules for many social circumstances. Patients mostly come from afar to the therapy centers. Distance, increased cost, physical exertion as well as lack of accompanying caregivers discourage them from choosing the weekly schedule. No study was carried out to compare the treatment outcome of these two regimens from our country's perspective. So, this study may aid in optimizing the neoadjuvant chemotherapy schedule in breast cancer in Bangladesh.

## Materials and methods

This quasi-experimental study was conducted from April 2022 to October 2023 at two centers in Dhaka, Bangladesh - the Department of Clinical Oncology, Bangabandhu Sheikh Mujib Medical University (BSMMU) and Department of Radiotherapy at the National Institute of Cancer Research and Hospital (NICR&H). Ethical approval was obtained from the Institutional Review Board of BSMMU (approval number BSMMU/2022/3993) before conducting the study. Informed consent was obtained from each patient before enrollment in the study. Data were collected using a pre-made questionnaire by face-to-face interviews with patients and from their investigation reports.

Patients

Female patients in stage-III (T0-2 N2 M0, T3-4 N1-2 M0, Any T N3 M0) breast cancer, according to the American Joint Committee on Cancer (AJCC) 8th edition [9], were enrolled in this study after histopathological confirmation, immunohistochemistry and staging workup during the mentioned period. Patients with early disease (e.g., T1 N0), metastasis (M1), Eastern Cooperative Oncology Group (ECOG) performance status of more than two, and patients who underwent surgery of the primary site (excluding diagnostic biopsy) were excluded. Disease progression, occurrence of unacceptable toxicity, and wish of the patients were criteria for discontinuation of treatment.

Intervention

Sixty-six patients were enrolled in the study and then divided equally into two arms (Arm A or three-weekly arm, and Arm B or weekly arm) by purposive sampling. Randomization could not be done mainly due to the weekly arm. Patients mostly came from a considerable distance from their homes to the therapy centers. Distance, increased cost, physical exertion as well as lack of accompanying caregivers discourage them from following the weekly schedule (described below). Patients of the weekly arm were mostly from inside Dhaka city or nearby. Distribution among the arms were primarily based on patients’ choice.

Patients in both arms were initially treated with doxorubicin (A) 60 mg/m^2^ intravenous (IV) on day 1 and cyclophosphamide (C) 600 mg/m^2^ IV on day 1 for four cycles [11]. Then, patients of arm A were treated by paclitaxel 175 mg/m^2^ by IV infusion over three hours three weekly for four cycles [11]. Patients in arm B received paclitaxel 80 mg/m^2^ by IV infusion over one hour weekly for 12 weeks [11]. All the patients in two arms received chemotherapy with all necessary pre-medications and other precautions. Patients got prefilled syringes of filgrastim 30 MIU subcutaneously when indicated. Toxicities were managed with conventional treatment and the patients were kept in close follow-up subsequently.

Assessment

Sixty-three patients completed their planned chemotherapy schedule. One patient in arm A and two in arm B didn’t continue treatment. Two patients in arm A and one in arm B were lost to follow-up. Ultimately, 60 patients, 30 in each arm, were analyzed. The patients were closely monitored during and after each cycle of chemotherapy. After four cycles, the first comprehensive follow-up with history, physical examination and relevant investigations were carried out to assess clinical response. At the end of the chemotherapy in both arms, a second comprehensive follow-up was carried out using history, physical examination and relevant investigations. ‘Response evaluation criteria for solid tumors’ (RECIST) version 1.1 was used to assess clinical response. Complete response, partial response, or no response was further defined according to the AJCC staging manual, 8th edition (table 48.1) [9]. Afterward, all patients underwent surgery, as indicated. Post-surgery pathological responses were assessed by analyzing histopathology reports. Acute toxicities (if present) were managed with conservative treatment. Data were analyzed according to the objectives of the study by using the SPSS (Statistical Package for Social Science) software program for Windows, version 26.0 (IBM Corp., Armonk, NY). The p-value less than 0.05 was taken as significant.

## Results

A total of 60 patients were analyzed for this study, 30 in each arm. Table 1 summarizes the baseline characteristics of patients in the two arms. Most patients were in the 41-50 years age group in both Arms. Patients with ECOG performance status up to 2 were included in this study. Most of the patients in both arms show an ECOG score of 1. Patients mostly had the left-sided disease, with the upper outer quadrant being the most common location of disease within the breast. Histologically, both ductal and lobular carcinomas were almost equally distributed in both arms, ductal carcinoma being the predominant one by far.

**Table 1:** Distribution of patients according to the baseline characteristics * Calculated using Student’s t-test or Chi-square (χ^2^) test ECOG = Eastern Cooperative Oncology Group, AJCC = American Joint Committee on Cancer, ER = Estrogen receptor, PR = Progesterone receptor

Variable	Arm A (n = 30)	Arm B (n = 30)	Total (n = 60)	p-value*
Age range (years)				0.513
30-40	4 (13.33%)	5 (16.67%)	9 (15%)	
41-50	12 (40%)	15 (50%)	27 (45%)	
51-60	10 (33.34%)	9 (30%)	19 (31.67%)	
61-70	4 (13.33%)	1 (3.33%)	5 (8.33%)	
ECOG performance status				0.4
0	5 (16.66%)	2 (6.67%)	7 (11.67%)	
1	14 (46.67%)	18 (60.0%)	32 (53.33%)	
2	11 (36.67%)	10 (33.33%)	21 (35.0%)	
Laterality of involved breast				0.417
Left	18 (60%)	21 (70%)	39 (65%)	
Right	12 (40%)	9 (30%)	21 (35%)	
Involved quadrant of breast				0.99
Upper-outer	18 (60%)	20 (66.67%)	38 (63.33%)	
Upper-inner	3 (10%)	3 (10%)	6 (10%)	
Lower-inner	5 (16.67%)	5 (16.67%)	10 (16.67%)	
Lower-outer	1 (3.33%)	0 (0.0%)	1 (1.67%)	
Central	3 (10%)	2 (6.66%)	5 (8.33%)	
Histology				0.774
Ductal	24 (80%)	22 (73.33%)	46 (76.67%)	
Lobular	5 (16.67%)	6 (20%)	11 (18.33%)	
Others	1 (3.33%)	2 (6.67%)	3 (5%)	
Histological differentiation (grade)				0.92
Well	5 (16.67%)	4 (13.33%)	9 (15%)	
Moderate	9 (30%)	10 (33.34%)	19 (31.67%)	
Poor	16 (53.33%)	16 (53.33%)	32 (53.33%)	
T stage (AJCC 8^th^ edition)				0.81
T1	0 (0%)	0 (0%)	0 (0%)	
T2	10 (33.33%)	9 (30%)	19 (31.67%)	
T3	15 (50%)	14 (46.67%)	29 (48.37%)	
T4	5 (16.67%)	7 (23.33%)	12 (20%)	
N stage (AJCC 8^th^ edition)				0.948
N0	7 (23.33%)	6 (20%)	13 (21.67%)	
N1	15 (50%)	14 (46.67%)	29 (48.33%)	
N2	7 (23.33%)	9 (30%)	16 (26.67%)	
N3	1 (3.34%)	1 (3.33%)	2 (3.33%)	
Stage group (AJCC 8^th^ edition)				0.678
IIIA	17 (56.67%)	19 (63.33%)	36 (60%)	
IIIB	9 (30%)	9 (30%)	18 (30%)	
IIIC	4 (13.33%)	2 (6.67%)	6 (10%)	
Hormone receptor status of tumor				0.559
ER and/or PR +ve	21 (70%)	23 (76.67%)	44 (73.33%)	
ER and PR both -ve	9 (30%)	7 (23.33%)	16 (26.67%)	
Proliferation index (Ki-67)				0.774
>20%	21 (70%)	22 (73.33%)	43 (71.67%)	
<20%	9 (30%)	8 (26.67%)	17 (28.33%)	

The most prevalent disease stage was IIIA. As per study criteria, no patients with early disease (e.g., T1), metastasis (M1) or ECOG performance status of more than 2 were included in the study. There were no statistically significant differences in terms of age, ECOG performance status, laterality, involved quadrant, histology, grading, staging, status of hormone receptor (ER/PR), or proliferation index (Ki-67) between the two arms (p-value > 0.05).

Table 2 shows the response of the primary tumor observed during the follow-up after the completion of all cycles of planned treatment. Complete clinical response (cCR) was more in Arm B (17 or 56.66%), while partial response (cPR) was more in Arm A (14 or 46.67%). In both of the arms, four (13.33%) patients had stable disease (cSD). No patient had progressive disease (cPD). The findings were not statistically significant (p>0.05).

**Table 2:** Clinical response observed after completion of all cycles of chemotherapy * Calculated using Chi-square (χ^2^) test

Response	Arm A (n = 30)	Arm B (n = 30)	Overall (n = 60)	p-value*
Complete response (cCR)	12 (40.00%)	17 (56.66%)	29 (48.33%)	0.377
Partial response (cPR)	14 (46.67%)	09 (30.00%)	23 (38.33%)	
Stable disease (cSD)	04 (13.33%)	04 (13.33%)	08 (13.33%)	
Progressive disease (cPD)	0	0	0	

**Figure 1:**
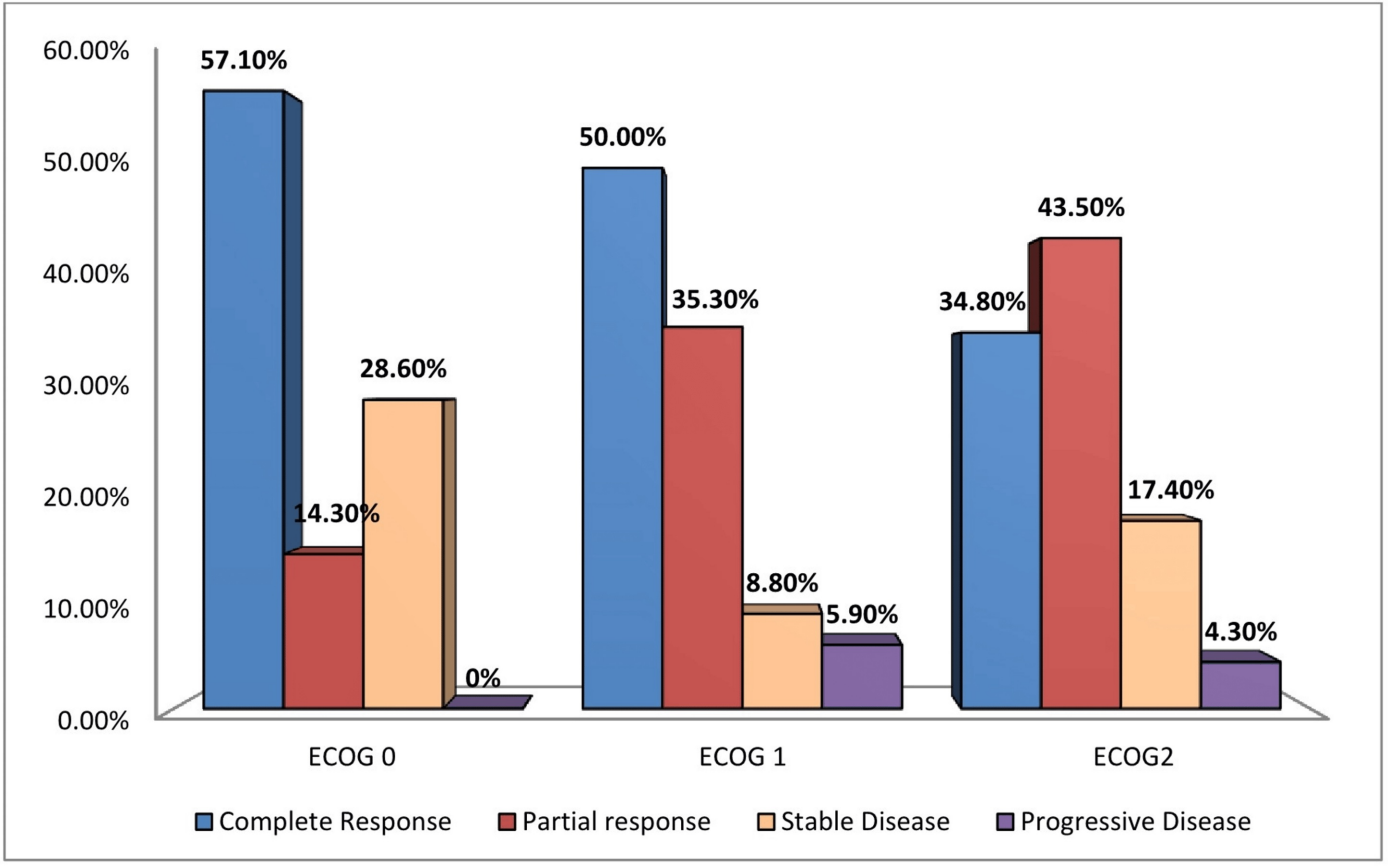
Clinical response observed at the end of all cycles of chemotherapy according to pre-treatment performance status (ECOG PS) ECOG PS = Eastern cooperative oncology group performance status

Figure 1 shows that a greater percentage of patients with pre-treatment ECOG PS-0 and ECOG PS-1 had achieved complete clinical response in comparison to patients with ECOG PS-2. However, the differences were not significant (p 0.60).

**Figure 2:**
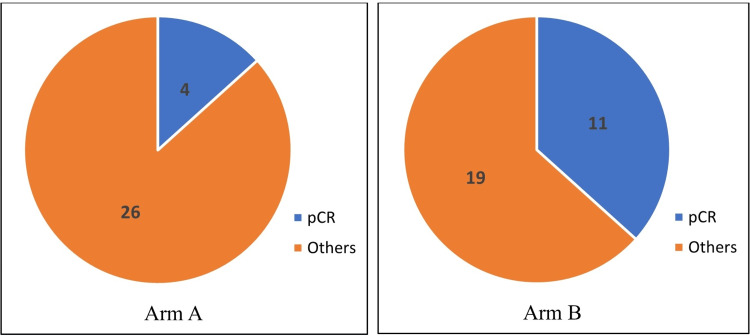
Status of pathological complete response (pCR) in both arms

Pathological complete responses in the two arms are compared in Figure 2. It shows that four (13.33%) patients in arm A and 11 (36.66%) in arm B had pCR. These 15 patients account for 25% of the total study patients. The difference was significant (p 0.037).

Table 3 shows pathological responses in each disease stage that were studied in both arms. In stage IIIA, 42.11% (eight patients out of 19) and 17.65% (three patients out of 17) patients had pCR in Arm B and A, respectively. Similarly, more patients in arm B (33.33% vs 11.11%) had pCR. None of the patients with clinical stage IIIC tumors had pCR. The observed differences were not significant (p>0.05).

**Table 3:** Status of pathological responses according to pretreatment staging of tumor pCR=pathological complete response; pPR= pathological partial response; NR=No response * Calculated using Chi-square (χ^2^) test or Fisher exact test

Stage	Response	Arm A	Arm B	Overall	p-value*
IIIA (n=36) (Arm A = 17 ; Arm B = 19)	pCR	03 (17.65%)	08 (42.11%)	11 (30.56%)	0.205
	pPR	08 (47.06%)	08 (42.11%)	16 (40.44%)	
	NR	06 (35.29%)	03 (15.79%)	09 (25.00%)	
IIIB (n=18) (Arm A = 9 ; Arm B = 9)	pCR	01 (11.11%)	03 (33.33%)	04 (22.22%)	0.519
	pPR	05 (55.56%)	04 (44.44%)	09 (50.00%)	
	NR	03 (33.33%)	02 (22.22%)	05 (27.78%)	
IIIC (n=6) (Arm A = 4 ; Arm B = 2)	pCR	0	0	0	0.10
	pPR	02 (50%)	0	02 (33.33%)	
	NR	02 (50%)	02 (100%)	04 (66.67%)	

**Figure 3:**
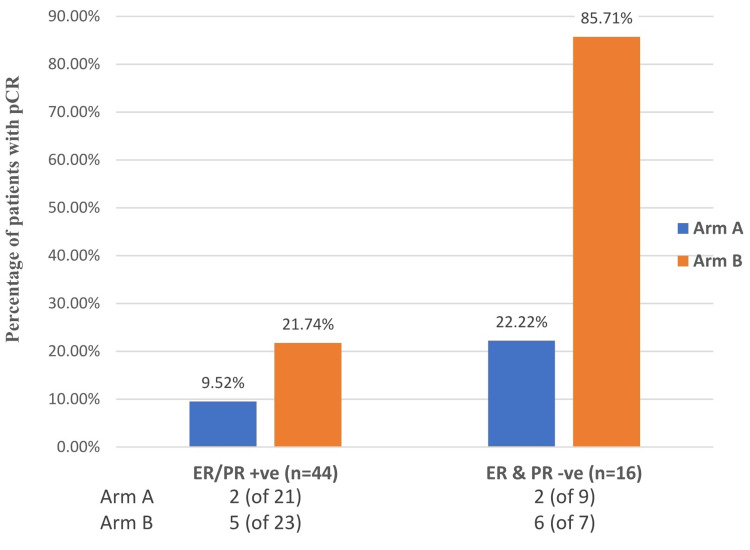
Status of pathological complete response (pCR) according to hormone receptor* status *All patients were human epidermal growth factor receptor 2 (HER2)-negative according to inclusion criteria; ER = Estrogen receptor, PR = Progesterone receptor.

Figure 3 shows that among 44 hormone receptor-positive patients (ER or PR or both), seven (15.91%) patients had complete pathological responses. The number was eight (50%) among 16 receptor-negative patients. Although more than 85% (six patients out of seven) of receptor-negative patients in arm B showed pCR, the observed differences were not significant (p>0.05).

Pathological responses according to histopathological grading in both arms are shown in Table 4. Pathological complete responses were more in arm B patients. Relatively more patients in arm A had no response. Observations were similar in all three histopathological grades. However, the observed differences were not significant (p>0.05).

**Table 4:** Status of pathological response according to histopathological grading pCR=pathological complete response; pPR= pathological partial response; NR=No response *Calculated using Chi-square (χ^2^) test or Fisher exact test

Histopathological grading	Response	Arm A	Arm B	Overall	p-value*
Well differentiated (n=9) (Arm A = 5 ; Arm B = 4)	pCR	01 (20%)	02 (50%)	03 (33.33%)	0.638
	pPR	02 (40%)	01 (25%)	03 (33.33%)	
	NR	02 (40%)	01 (25%)	03 (33.33%)	
Moderately differentiated (n=19) (Arm A = 9 ; Arm B = 10)	pCR	02 (22.22%)	05 (50%)	07 (36.84%)	0.42
	pPR	05 (55.56%)	03 (30%)	08 (42.11%)	
	NR	02 (22.22%)	02 (20%)	04 (21.05%)	
Poorly differentiated (n=32) (Arm A = 16 ; Arm B = 16)	pCR	01 (6.25%)	04 (25%)	05 (15.63%)	0.26
	pPR	10 (62.25%)	08 (50%)	18 (56.25%)	
	NR	05 (31.25%)	04 (25%)	09 (28.12%)	

Table 5 shows pathological responses according to the Ki-67 proliferation index in both arms. Pathological complete responses were higher in arm B patients while more arm A patients had no response. However, the observed differences were not significant (p>0.05).

**Table 5:** Status of pathological response according to Ki-67 proliferation index pCR=pathological complete response; pPR= pathological partial response; NR=No response * Calculated using chi-square (χ^2^) test or Fisher exact test

Ki-67	Response	Arm A	Arm B	Overall	p-value*
Ki-67>20% (n=43) (Arm A = 21 ; Arm B = 22)	pCR	04 (19.05%)	10 (45.45%)	14 (32.56%)	0.174
	pPR	15 (71.43%)	11 (50.00%)	26 (60.47%)	
	NR	02 (9.52%)	01 (4.55%)	03 (6.97%)	
Ki-67<20% (n=17) (Arm A = 9 ; Arm B = 8)	pCR	0	01 (12.50%)	01 (05.88%)	0.8
	pPR	02 (22.22%)	02 (25.00%)	04 (23.53%)	
	NR	07 (77.78%)	05 (62.50%)	12 (70.59%)	

## Discussion

Sixty patients with HER2-negative stage III breast cancer were analyzed in the present study. The majority of the patients were in 41-50 years of age followed by 51-60 years in both arms. In Arm A, 12 (40%) patients were in the 41-50 years group, whereas 15 (50%) patients in Arm B were in that group category. These observations closely correlate with the findings of the Cancer registry report (2015-2017) of the National Institute of Cancer Research and Hospital (NICR&H), as well as other studies [6,12,13]. About 47% (14) of patients in Arm A and 60% (18) in Arm B had ECOG performance status 1. Since most of the patients of Arm B were in this group, this could explain their tolerability of systemic treatment thereafter. More patients had cancer on the left side (65% vs 35%) in this study. Although cancer is somewhat more common in the left than in the right breast, Fatima et al. mentioned larger breast size, unilateral lactation detection, bias due to dominant right-handedness, and denser left breast as possible factors in their study [14,15].

The majority of the patients were in stage IIIA, 17 (56.67%) patients in Arm A, and 19 (63.33%) patients in Arm B. Most patients (44 or 73%) were hormone receptor (HR) positive (ER and/or PR), whereas about 26% of the patients were triple negative. These findings are supported by Yin et al. [16]. The Ki-67 proliferative index of breast cancer is considered an important predictor of outcome after treatment [10,17]. In this study, 21 (70%) patients in Arm A and 22 (73.33%) patients in Arm B presented with Ki-67 more than 20%. The prognostic role of reduction in Ki-67 level after neoadjuvant chemotherapy is evaluated by Jones et al., but the discussion is beyond the focus of the present study [18].

Clinical response evaluation was done periodically with history, relevant physical examination and investigations according to the follow-up schedule which was set earlier. The first comprehensive follow-up was conducted after the fourth cycle of completion of chemotherapy with AC. The second comprehensive follow-up was conducted after the completion of all chemotherapy and before surgical management. Clinical complete response (cCR) was more in Arm B (56.66% vs 40%). Clinical partial response (PR) was 14 (46.67%) in Arm A and 09 (30.00%) in Arm B. Four (13.33%) patients had stable disease (SD) in both arms. No patient in both arms had progressive disease. Although the findings matched with the Green et al. study, they were not statistically significant (p 0.377) [17]. More than 85% of patients had a response to neoadjuvant chemotherapy, either in the form of CR or PR, with less than 15% of patients being non-responders clinically.

Clinical response was predominantly found in patients with ECOG scores 0 and 1 in both arms. Although the difference was not statistically significant, the complex interrelationship between tumor response to therapy and body immunity can be a probable reason behind this finding.

The patients were assessed after a definitive surgical procedure. Pathological complete response (pCR) was achieved in 15 (25%) patients. It was 13.33% (four patients) in Arm A and 36.66% (11 patients) in Arm B. This difference was statistically significant (0.037). A more frequent administration of paclitaxel may have coincided favorably with the timing of pathophysiologic responses to the drug in the tumor. We have to recognize the fact that weekly administration employs a higher dose of paclitaxel, which has the potential to result in higher toxicities, at least in theory. As pCR can be a surrogate for overall survival, the advantages of weekly paclitaxel may outweigh the effects of a higher dose. These findings are similar to the studies done by Green et al. [17]. There was a noticeable decline in the number of patients with the complete response from before surgery to after in both arms (from 12 to 4 in Arm A and from 17 to 11 in Arm B), i.e., pCR was much less than cCR. This reflects the inherent limitations of clinical methods in identifying residual cancers after neoadjuvant treatment.

The pCR was also compared against the pretreatment disease stage, hormone receptor status, grade, and proliferation index. Patients with stage IIIC tumors had more progressive disease. More patients in stages IIIA and IIIB had pCR in Arm B (42.11% vs 17.65% and 33.33% vs 11.11%, respectively), while pPR was similar in both arms. A similar observation was mentioned by Miglietta et al. [19]. None of the patients with clinical stage IIIC tumors had pCR. The observed differences were not significant (p>0.05). Patients with higher stages are always unlikely to have a complete response. Moreover, as patients with all stages were not included in the study, no definite conclusions can be drawn. Further studies with larger sample sizes are needed in this regard.

According to study criteria, only HER2-negative patients were included. So, they were either ER and/or PR-positive or triple-negative patients. It was found that ER and/or PR-positive tumors had a lower rate of pCR in comparison to receptor-negative (triple-negative) tumors in both arms. In receptor-positive tumors, pCR were 9.52% (two patients out of 21) and 21.74% (five patients out of 23) in Arm A and Arm B, respectively. Whereas in the case of triple-negative disease, Arm A had 22.22% (two patients out of nine) pCR and Arm B had 85.71% (six patients out of seven) pCR. This finding correlates with Mazouni et al. [20]. However, the p-value was not significant (p 0.33). Such a high response in Arm B was probably coincidental. There were only eight patients in Arm B with triple-negative disease, and seven of them achieved pCR. This needs further systematic evaluation using a larger sample size before any conclusion can be drawn.

It was observed that pCR was predominant in a high Ki-67 proliferation index, particularly in Arm B (45.45% vs 19.05%). Overall, these findings were similar to those of the Miglietta et al. study [19]. Resende et al. reported that patients with Ki-67 >20% had pCR 41.9% and it was 11.6% in patients with Ki-67 <20%, which nearly co-relates with this study [21].

There were multiple limitations of this study despite taking meticulous care. As it was a non-randomized study, selection bias was present. This makes interpretation of the observed differences difficult. For example, a better pCR rate in the weekly-paclitaxel arm can be due to some unknown factor that was not accounted for. Evaluation of the toxicity profile could not be done due to poor logistical support available. The statistically significant difference in pCR might not translate into a clinically significant advantage considering toxicity. We intend to work on this in the future. Consideration of toxicity is bound to play a key role in selecting the appropriate therapy regimen. The time period was short for evaluating the outcomes like progression-free survival or overall survival. Patients were sampled from two centers in Dhaka city. So, it doesn’t reflect the nationwide scenario. Further multi-center studies with larger sample sizes over longer time periods with stratification should be carried out.

## Conclusions

This study aimed to look at the treatment response of four cycles of three-weekly administrations of AC followed by paclitaxel weekly or every three weeks in a neoadjuvant setting in HER2-negative, stage III breast cancer patients. The weekly schedule was found to have a better pathological response rate than the three-weekly one. However, the limitations of the study necessitate further systematic evaluation. The toxicity profile may dictate the choice between two regimens in individual patients. Further studies with larger sample sizes, along with analysis of toxicities, are needed.
